# Estimating SARS‐CoV‐2 Omicron XBB.1.5 Spike‐Directed Functional Antibody Levels From an Anti‐Receptor Binding Domain Wuhan‐Hu‐1‐Based Commercial Immunoassay Results

**DOI:** 10.1002/jmv.70130

**Published:** 2025-01-15

**Authors:** Ángela Sánchez‐Simarro, Enric Cuevas‐Ferrando, Daniel Fernández‑Soto, Brayan Grau, Eliseo Albert, Estela Giménez, Ana Isabel Avilés‑Alía, Luciana Rusu, Ron Geller, Hugh T. Reyburn, David Navarro

**Affiliations:** ^1^ Microbiology Service Clinic University Hospital, INCLIVA Health Research Institute Valencia Spain; ^2^ Department of Immunology and Oncology National Centre for Biotechnology, CNB‐CSIC Madrid Spain; ^3^ Institute for Integrative Systems Biology (I2SysBio) Universitat de Valencia‐CSIC Valencia Spain; ^4^ Department of Microbiology School of Medicine, University of Valencia Valencia Spain; ^5^ CIBER de Enfermedades Infecciosas Instituto de Salud Carlos III Madrid Spain

**Keywords:** anti‐RBD total antibodies, neutralizing antibodies, NK cells, Omicron XBB.1.5, Random Forest modeling, Roche Elecsys® anti‐SARS‐CoV‐2 S assay, SARS‐CoV‐2

## Abstract

We investigated whether antibody concentrations measured in plasma using the Roche Elecsys® Anti‐SARS‐CoV‐2 S assay (targeting the receptor binding domain, RBD) could estimate levels of Wuhan‐Hu‐1 and Omicron XBB.1.5 spike‐directed antibodies with neutralizing ability (NtAb) or those mediating NK‐cell activity. We analyzed 135 plasma samples from 39 vaccinated elderly nursing home residents. A strong correlation was found for NtAb against both Wuhan‐Hu‐1 (Rho = 0.73, *p* < 0.001) and Omicron XBB.1.5 (sub)variants (Rho = 0.73, *p* < 0.001). Moderate positive correlations were observed for NK‐cell activity, based on lysosome‐associated membrane protein 1 (LAMP1)‐producing NK cells stimulated with Wuhan‐Hu‐1 (Rho = 0.43, *p* < 0.001) and Omicron XBB.1.5 spike proteins (Rho = 0.50, *p* < 0.001). Similarly, interferon‐gamma (IFN‐γ)‐producing NK‐cell frequencies showed moderate correlations (Wuhan‐Hu‐1: Rho = 0.43, *p* < 0.001; Omicron XBB.1.5: Rho = 0.50, *p* < 0.001). Random Forest models accurately predicted NtAb levels against Wuhan‐Hu‐1 (*R* ^2^ = 0.72), though models for Omicron XBB.1.5 were less robust. Anti‐RBD antibody concentrations of 4.73 and 5.02 log_10_ BAU/mL predicted high NtAb levels for Wuhan‐Hu‐1 and Omicron XBB.1.5, respectively. Antibody thresholds for predicting functional NK cell‐mediated responses were 4.73 log_10_ and 4.54 log_10_ BAU/mL for Wuhan‐Hu‐1 and Omicron XBB.1.5, respectively. For LAMP1‐producing NK cells, the thresholds were 4.94 and 4.75 log_10_ BAU/mL for Wuhan‐Hu‐1 and Omicron XBB.1.5, respectively. In summary, total anti‐RBD antibody levels measured by the Roche assay may allow inference of NtAb levels and, to a lesser extent, Fc‐mediated NK‐cell responses against Omicron XBB.1.5.

## 1 Introduction

Protection against SARS‐CoV‐2 infection and developing severe forms of COVID‐19 likely involves innate and adaptive immune responses [1, 2, 3]. Antibodies recognizing the SARS‐CoV‐2 spike (S) protein may display a variety of functional properties, such as virus neutralization, critically contributing to the clearance of free circulating virions, or mediation of Fc‐dependent immune cell activation, for example, the lysis of infected cells by NK cells (antibody‐dependent cell cytotoxicity‐ADCC) [4, 5, 6]. Importantly, while the efficiency of pre‐existing neutralizing antibodies (NtAb) against emerging SARS‐CoV‐2 subvariants that accumulate non‐conservative mutations within the S protein is dramatically compromised [7, 8, 9], antibodies mediating NK‐cell responses appear to be more cross‐reactive, thus maintaining an acceptable activity against NtAb‐escape SARS‐CoV‐2 mutants [10, 11, 12, 13, 14, 15]. Quantitation of functional SARS‐CoV‐2 S‐directed antibodies in sera could be informative in assessing the effectiveness of adapted COVID‐19 vaccines [16]. Unfortunately, NtAb assays using either S‐pseudotyped virions, recombinant viruses, or live SARS‐CoV‐2 strains, and ADCC assays are technically demanding and thus inaccessible to most laboratories. Several commercially available quantitative chemiluminescent immunoassays measure antibody levels against the Wuhan‐Hu‐1 S protein or the S receptor binding domain (RBD). Prior studies showed that antibody concentrations measured by these immunoassays tend to correlate with NtAb levels directed against the ancestral strain, although the degree of correlation varies widely across assays [17, 18, 19]. Here, we used different approaches, including Random Forest modeling [20], to explore the potential of the Roche Elecsys® Anti‐SARS‐CoV‐2 S assay (Roche Diagnostics, Pleasanton, USA), that allows quantitation of total antibodies targeting the RBD of the Wuhan‐Hu‐1 S protein, to infer NtAb titers and antibody Fc‐mediated NK‐cell frequencies against the heterologous SARS‐CoV‐2 subvariant Omicron XBB.1.5. This variant of concern (VoC) was circulating when the samples used in our analyses were collected from a cohort of elderly nursing home residents, who frequently develop severe COVID‐19, and who completed a regular COVID‐19 vaccination schedule and received two vaccines (Wuhan‐Hu‐1‐based) booster doses. If it were possible to infer the magnitude of functional antibody responses using widely available commercial assays this would greatly facilitate assessing the risk of severe SARS‐CoV‐2 infection and thus permit precision booster vaccination schedules.

## 2 Materials and Methods

### 2.1 Study Population

A total of 135 plasma specimens from 39 elderly nursing home residents (31 females and 8 males; median age, 91 years; range: 66–103) enrolled between August 2021 and October 2022 were available for analysis. Clinical characteristics of participants and data returned by the different immunoassays specified below were previously reported [15]. All individuals had completed regular vaccination with mRNA‐licensed vaccines (Spikevax® or Comirnaty®). Plasma was collected before and after one or two booster vaccine (mRNA) doses. Importantly, the percentage of participants who had contracted SARS‐CoV‐2 infection, according to the presence of anti‐SARS‐CoV‐2‐Nucleocapsid IgG in plasma (Roche Elecsys® assay) and/or a record of a positive rapid antigen or SARS‐CoV‐2 PCR assay, varied over time. Before receipt of the first booster vaccine dose, there were 27 SARS‐CoV‐2‐naïve participants, whereas only eight participants remained SARS‐CoV‐2‐naïve after receiving the second booster dose. Twenty‐one participants were (re)infected by pre‐XBB.1.5 Omicron subvariants [15]. The current study was carried out under the epidemiological surveillance competencies of the Valencia Government Health Department (Law 16/2003/May 28 on Cohesion and Quality of the National Health System, and Law 10/2014/December 29 on Public Health of the Valencian Community), without requiring informed consent, as approved by the institutional ethical review board of the Fundación para el Fomento de la Investigación Sanitaria y Biomédica de la Comunitat Valenciana FISABIO, (Valencia, Spain). Likewise, according to local law and regulations, the publication of the data is exempt from the approval of a research ethics committee. Personal data from nursing homes and residents were processed according to European data protection regulations. All methods were performed in accordance with the Declaration of Helsinki and the Belmont Report guidelines and regulations.

### 2.2 SARS‐CoV‐2 Immunoassays

All procedures in the current study were carried out as previously reported [15]. Briefly, quantitation of total antibodies targeting the RBD of the SARS‐CoV‐2 Wuhan‐Hu‐1 spike (S) protein was conducted using the Roche Elecsys® Anti‐SARS‐CoV‐2 S assay (Roche Diagnostics, Pleasanton, USA). NtAbs specific to the S protein were measured utilizing a green fluorescent protein (GFP)‐expressing vesicular stomatitis virus system, pseudotyped with either the Wuhan‐Hu‐1 or XBB.1.5 subvariants [15]. The limit of detection of this assay was reciprocal IC_50_ of 20. The assay for antibody‐induced natural killer (NK) cell activation was conducted according to a previously reported protocol [15] using recombinant S proteins representative of SARS‐CoV‐2, either the Wuhan‐Hu‐1 or Omicron XBB.1.5 variants (SinoBiological). Frequencies of NK‐cell‐producing lysosome‐associated membrane protein 1 (LAMP1) or interferon‐γ (IFN‐γ) were determined by flow cytometry (Cytoflex flow cytometer; Beckman Coulter).

### 2.3 Statistical Analyses

Correlation analyses employed Spearman's rank correlation coefficient. The reported *p* value*s* are two‐sided and exact; a *p*‐value of less than 0.05 was deemed to indicate statistical significance. Random Forest analysis was used to predict immune response parameters from total antibody values provided by the Roche anti‐RBD assay. Random Forest models with 2000 trees (ntree = 2000) were trained using 90% of the data, with the remaining 10% used for model testing. As a cross‐validation method, training and testing were repeated five times, resulting in five Random Forest models for each predicted immune response parameter. For each iteration, the model's accuracy was assessed by computing the root mean squared error (RMSE) and the mean absolute error (MAE) to evaluate the dispersion of errors between the predicted and actual data: where ŷ1, ŷ 2, … ŷn are predicted values; y1, y2, … yn are actual values; and n is the number of observations. Normalization of RMSE (NRMSE) and MAE (NMAE) for model comparison between target variables was performed as follows:

$$\mathrm{RMSE}=\sqrt{\sum_{i=1}^{n}\frac{{\left({ŷ}_{i}-y_{i}\right)}^{2}}{n}}\mathrm{MAE}=\frac{1}{N}\;\sum_{i=1}^{N}\left|y_{i}-{ŷ}_{i}\right|,$$

$$\mathrm{NRMSE}=\frac{\mathrm{RMSE}}{Y_{\max}-Y_{\min}}\mathrm{NMAE}=\frac{\mathrm{MAE}}{Y_{\max}-Y_{\min}},$$

where Y_max_ and Y_min_ are the maximum and minimum values of the predicted variable in the dataset, respectively. Also, logistic regression models were fitted to the Random Forest average prediction results by using the “glm” function (from the “stats” package in R) to approximate the overall variance explained by the model. Additionally, logistic regression models with a binomial family were used to estimate the probability that immune response parameters exceed specific thresholds of detectability and high functionality, based on total anti‐SARS‐CoV‐2 RBD antibody levels. All statistical analyses were executed by using GraphPad Prism version 8.4.3. (GraphPad Software Inc., La Jolla), SPSS version 20.0 (IBM SPSS Statistics), STATA version 17.0 (StataCorp), and R version 4.3.3. Script for data processing is available at https://github.com/enriccf/Anti-S-NtAb/blob/main/scriptR_AntiS_NtAb.txt.

## 3 Results

### 3.1 Correlation Between Anti‐SARS‐CoV‐2 RBD Total Antibody Concentrations as Quantitated By the Roche Elecsys Assay and SARS‐CoV‐2 Spike‐Binding Functional Antibody Levels

**Figure 1:**
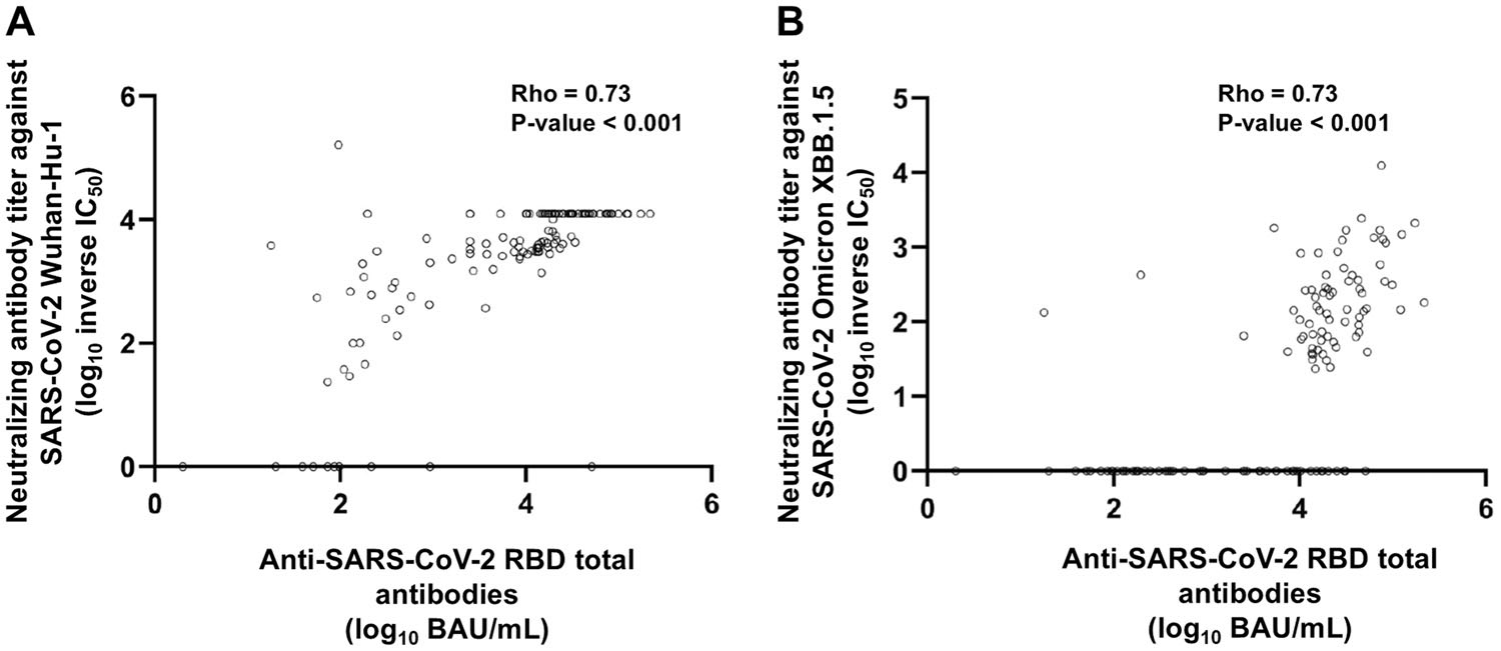
Correlation between anti‐RBD total antibodies measured by the Roche Elecsys® Anti‐SARS‐CoV‐2 S assay (in BAU/mL) and neutralizing antibody titers (log_10_ inverse IC_50_) against (A) Wuhan‐Hu‐1 and (B) Omicron XBB.1.5. Rho and *p‐*values (Spearman's Rank test) are shown.

**Figure 2:**
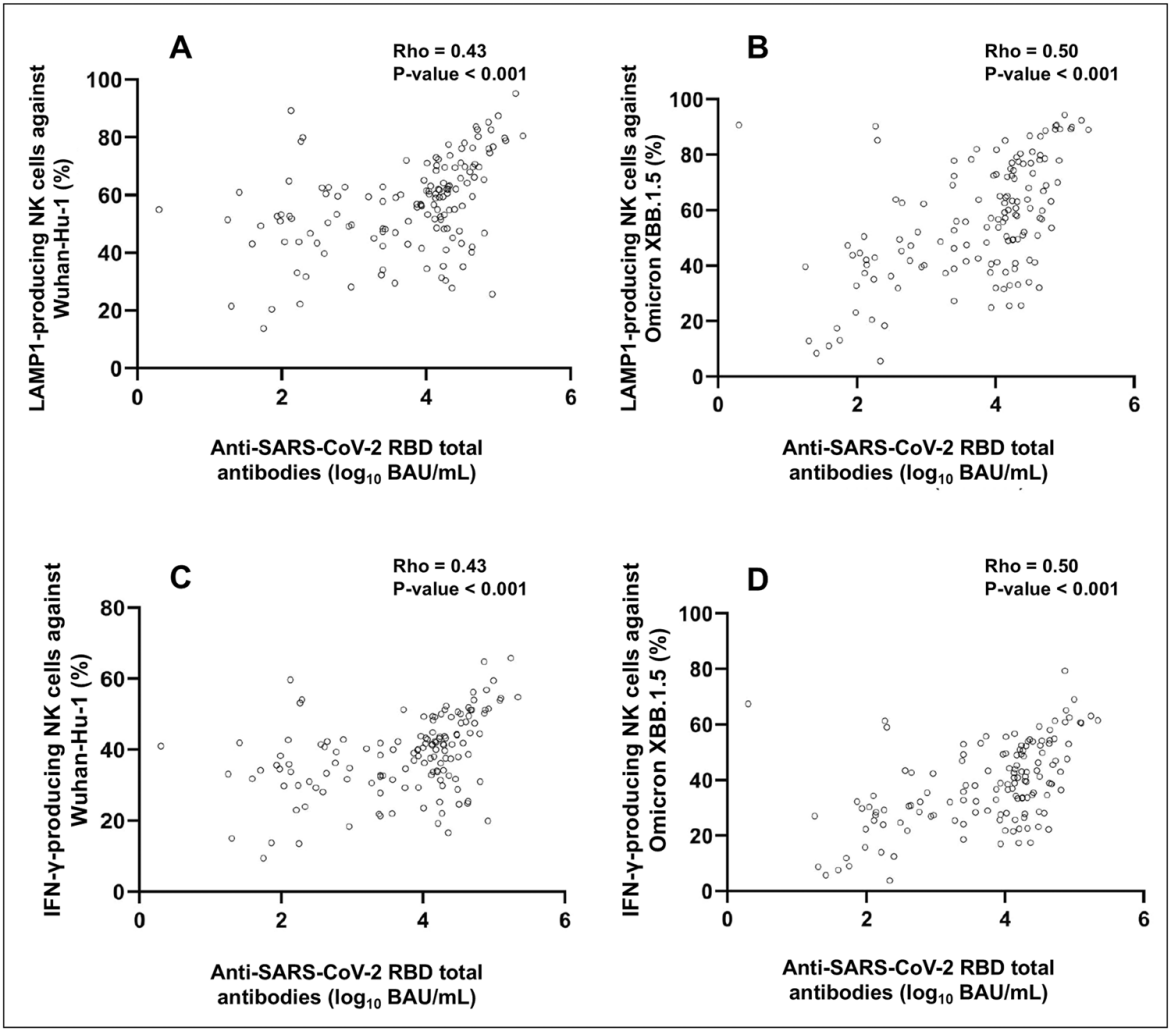
Correlation between anti‐RBD total antibodies measured by the Roche Elecsys® Anti‐SARS‐CoV‐2 S assay (in BAU/mL) and LAMP1‐producing and IFN‐γ‐producing NK‐cell frequencies upon stimulation with Wuhan‐Hu‐1 (A and C) or Omicron XBB.1.5 (B and D) Spike proteins. Rho and *p*‐values (Spearman's Rank test) are shown.

Due to the data distribution, we used the non‐parametric Spearman's rank test to assess the degree of correlation between anti‐RBD total antibody concentrations and Wuhan‐Hu‐1 and Omicron XBB.1.5 S‐directed functional antibodies. Overall, moderate‐to‐strong correlations were observed between anti‐RBD total antibody concentrations (in BAU/mL) and immune parameters assessed, ranging from Rho = 0.43–0.74 (*p* values < 0.001). Interestingly, a strong positive correlation was seen for NtAb (inverse IC_50_) against both Wuhan‐Hu‐1 (Rho = 0.73, *p* < 0.001) and Omicron XBB.1.5 (sub)variants (Rho = 0.73, *p* < 0.001) (Figure 1A,B, respectively). In contrast, moderate positive correlations were observed for LAMP1‐producing NK‐cell frequencies upon stimulation with Wuhan‐Hu‐1 (Rho = 0.43, *p* < 0.001) and Omicron XBB.1.5 S proteins (Rho = 0.50, *p* < 0.001) (Figure 2A,B, respectively). Similarly, moderate correlations were noticed for IFN‐γ‐producing NK‐cell frequencies (Wuhan‐Hu‐1; Rho = 0.43; *p* < 0.001 and Omicron XBB.1.5; Rho = 0.50; *p* < 0.001) (Figure 2C,D).

### 3.2 Predictive Modeling of Functional Antibody Levels From Total Anti‐SARS‐CoV‐2 RBD Antibody Concentrations

Random Forest predictive models based on linear regression analyses were built and trained to assess the extent to which total antibody values provided by the Roche RBD assay could predict the magnitude of antibody‐mediated functional immune responses and average and best (N)RMSE and (N)MAE values calculated (Table 1). The average NRMSE and NMAE values for each predicted immune response ranged from 0.16 (Omicron XBB.1.5‐specific NtAb levels) to 0.24 (Wuhan‐Hu‐1‐specific NtAb levels) and from 0.08 (Omicron XBB.1.5‐specific NtAb levels) to 0.22 (Wuhan‐Hu‐1 antibody‐mediated LAMP‐1‐producing NK‐cell frequencies), respectively. As an extra validation method, linear regression models were fitted to the Random Forest average predictions for NtAb titers and antibody‐mediated NK‐cell frequencies to obtain equations predicting functional antibody levels (Table 1, Supporting Information S1: Figures S1–6). The linear regression models extracted presented *R* ^2^ values ranging from 32% (Omicron XBB.1.5‐specific NtAb levels) to 72% (Wuhan‐Hu‐1‐specific NtAb levels).

**Table 1:** Performance of Random Forest models (average and best, *n* = 5) in predicting variant‐specific NtAb titers and NK‐cell‐mediated responses against Wuhan‐Hu‐1 and Omicron XBB.1.5 variants based on total anti‐RBD antibody concentrations measured using the Roche Elecsys® Anti‐SARS‐CoV‐2 S assay. Fitted logistic regression models with predictive equations and *R* ^2^ values are also provided for each immune response prediction. RMSE: Root Mean Square Error; NRMSE: Normalized RMSE; MAE: Mean Absolute Error; NMAE: Normalized MAE.

SARS‐CoV‐2 variant	Immune response	Average (*n* = 5 Random Forest models)		Best Random Forest		Fitted logistic regression model	*R* ^2^
		RMSE/NRMSE	MAE/NMAE	RMSE/NRMSE	MAE/NMAE		
	Specific NtAb (log_10_ inverse IC_50_)	2981.35/0.24	2255.99/0.18	1715.40/0.14	1390.08/0.11	*y* = −4064.22 + 2805.67 × total anti‐RBD	0.72
Wuhan‐Hu‐1	Ab‐mediated‐LAMP1 (%)	15.47/0.27	12.22/0.22	13.1/0.23	9.95/0.18	*y* = 41.14 + 3.80 × total anti‐RBD	0.48
	Ab‐mediated‐IFN‐γ (%)	10.87/0.19	8.4/0.15	9.42/0.16	7.94/0.12	*y* = 24.93 + 3.33 × total anti‐RBD	0.46
	Specific NtAb (log_10_ inverse IC_50_)	390.69/0.16	200.39/0.08	89.19/0.04	61.07/0.02	*y* = −142.45 + 78.96 × total anti‐RBD	0.32
Omicron XBB.1.5	Ab‐mediated‐LAMP1 (%)	19.56/0.22	15.9/0.18	14.06/0.16	12.57/0.14	*y* = 30.72 + 5.87 × total anti‐RBD	0.41
	Ab‐mediated‐IFN‐γ (%)	13.42/0.18	11.15/0.15	11.33/0.15	9.33/0.12	*y* = 20.52 + 4.42 × total anti‐RBD	0.64

For SARS‐CoV‐2 variant‐specific NtAbs, MAEs, indicating the average absolute errors between predicted and observed values in the dataset, were 3.35 log_10_ and 2.30 log_10_ (inverse IC_50_ titer) for the Wuhan‐Hu‐1 and XBB.1.5 variants, respectively. Taking into account that the maximum value within NtAb measurements was 4.09 and 3.39 log_10_ (inverse IC_50_ titer), the average model absolute error for the prediction of NtAb levels against the Wuhan‐Hu‐1 variant accounted for 18% of the maximum value, while for the Omicron XBB.1.5 variant, it was much lower, 8%. However, the models explained the variance of the data much better (*R* ^2^ = 0.72) for the Wuhan‐Hu‐1 variant than for Omicron XBB.1.5 (*R* ^2^ = 0.32). As for Fc‐mediated NK‐cell responses, MAEs were 8.4% and 11.15% for IFN‐γ‐producing NK‐cell frequencies against Wuhan‐Hu‐1 and Omicron XBB.1.5 (sub)variants, respectively, and 12.22% and 15.9% for LAMP1‐producing NK‐cell frequencies against Wuhan‐Hu‐1 and Omicron XBB.1.15 (sub)variants, respectively (Table 1, Supporting Information S1: Figures S1–6).

### 3.3 Total Anti‐SARS‐CoV‐2 RBD Antibody Concentrations Predicting Detectable, High, and Functional Antibody Levels

**Figure 3:**
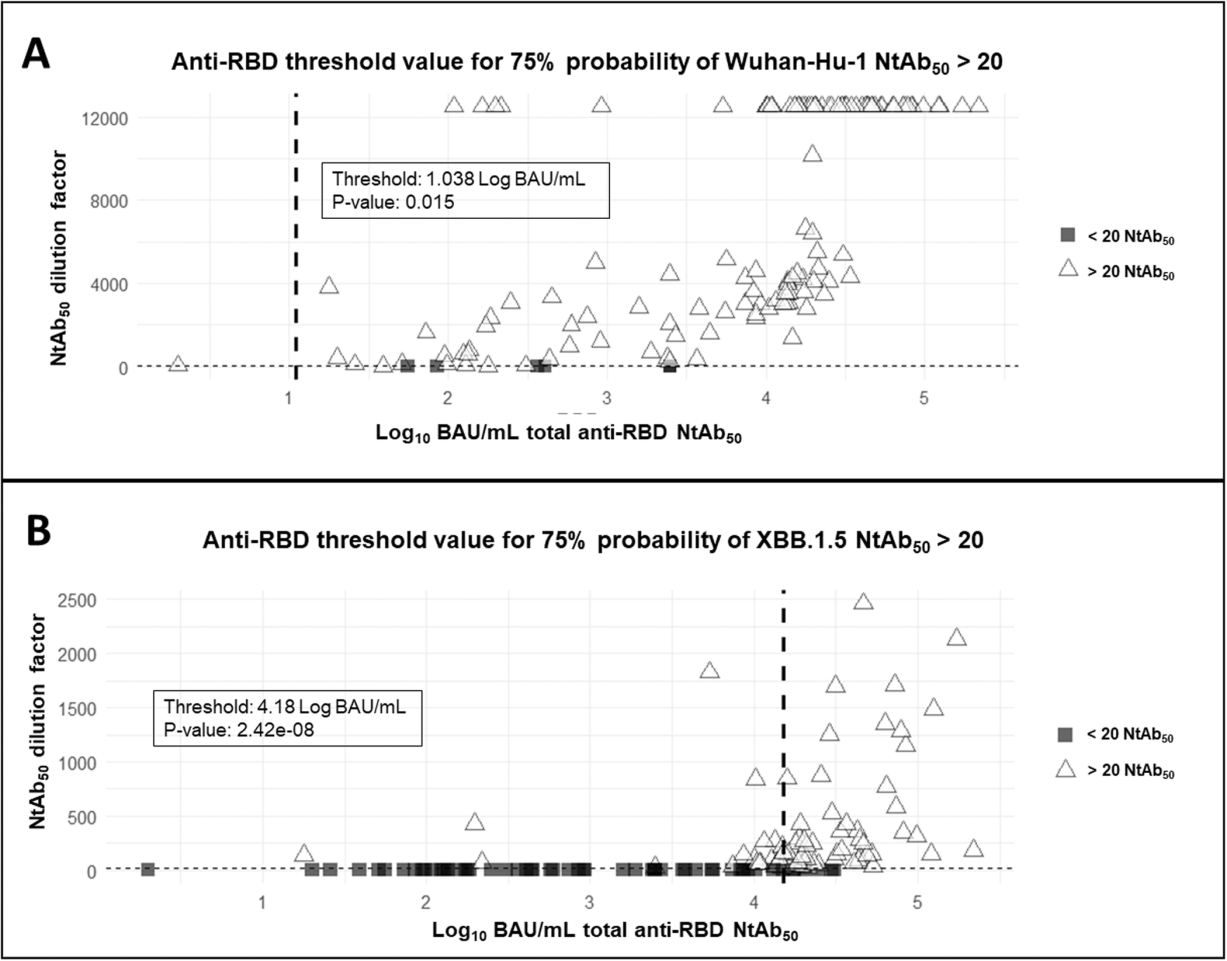
Threshold values for anti‐RBD total antibodies (in log_10_ BAU/mL) predicting detectable (limit of detection inverse of 20 IC_50_) neutralizing antibody (NtAb) titers against Wuhan‐Hu‐1 (A) and Omicron XBB.1.5 (B) with a probability > 75%. *p*‐values are shown.

**Figure 4:**
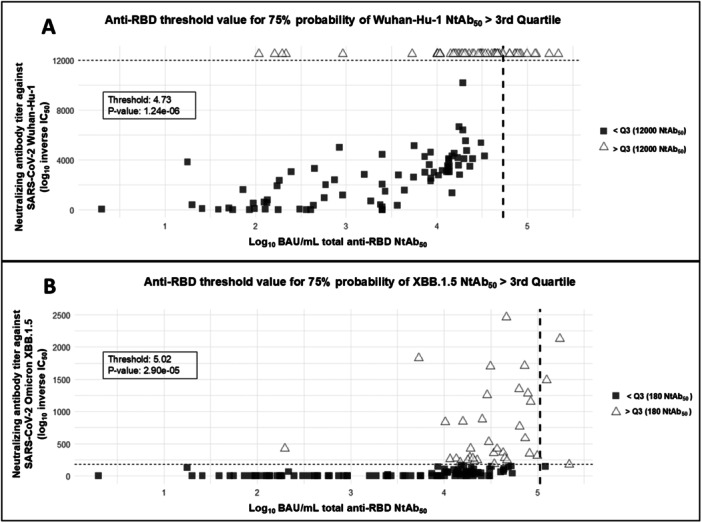
Threshold values for anti‐RBD total antibodies (in log_10_ BAU/mL) predicting threshold values for total anti‐RBD NtAb (Log BAU/mL) values that can be translated into a > 75% probability of predicting a high neutralizing antibody response (in NtAb_50_ dilution factor) in Wuhan‐Hu‐1 and XBB.1.5 SARS‐CoV‐2 variants. The vertical dashed line corresponds to the threshold value; the horizontal dashed line represents the third Quartile value from *n* = 135 plasma samples from *n* = 39 elderly nursing home residents.

**Figure 5:**
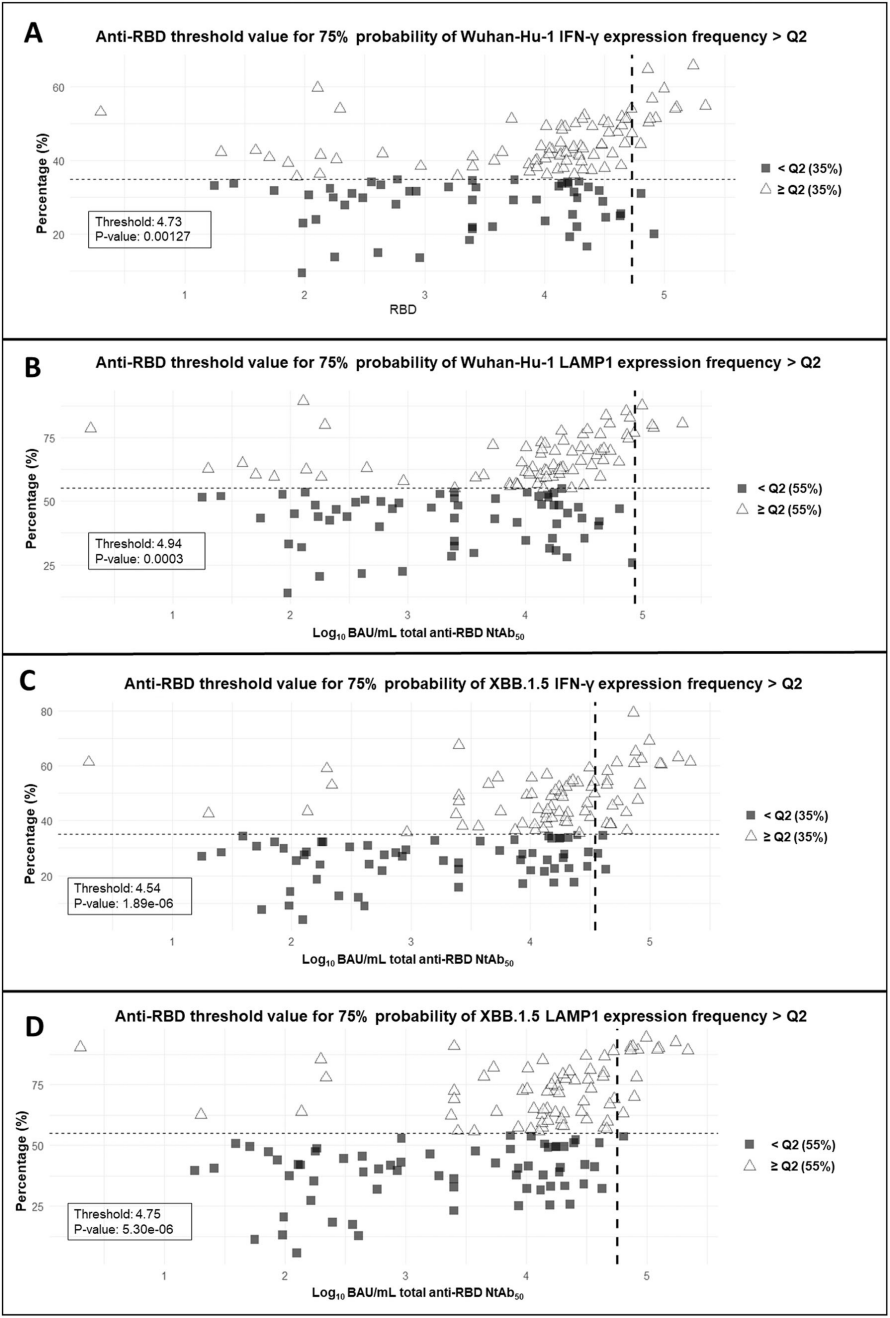
Threshold values for total anti‐RBD NtAb (Log BAU/mL) values that can be translated into a > 75% probability of predicting a functional antibody Fc‐mediated NK‐cell response (in IFN‐γ and LAMP1 producing NK‐cell expression frequencies) against Wuhan‐Hu‐1 and XBB.1.5 SARS‐CoV‐2 variants. The vertical dashed line corresponds to the threshold value; the horizontal dashed line represents the second Quartile value from *n* = 135 plasma samples from *n* = 39 elderly nursing home residents.

Next, anti‐RBD total antibody threshold values that predicted detectable NtAb levels with a > 75% probability were determined by using logistic regression models. The figures were 1.04 (*p* < 0.005) and 4.18 (*p* < 0.005) log_10_ BAU/mL for Wuhan‐Hu‐1 and Omicron XBB.1.5, respectively (Figure 3). This analysis could not be performed for NK‐cell frequencies as all samples returned detectable responses. We also defined anti‐RBD total antibody titers predicting high‐level functional antibody responses against both SARS‐CoV‐2 variants. We arbitrarily defined high‐level responses as those equal to or above the third quartile (≥ Q3). The total anti‐RBD antibody concentrations at which there was a > 75% probability of obtaining high NtAb titers were 4.73 (*p* < 0.0001) and 5.02 log_10_ BAU/mL (*p* < 0.0001) for Wuhan‐Hu‐1 and Omicron XBB.1.5, respectively (Figure 4A,B). Contrarily, no thresholds could be established for antibody‐mediated NK‐cell frequencies under these analysis settings (prediction of levels ≥ Q3 at 75% probability). Nevertheless, anti‐RBD total antibody thresholds could be determined for predicting antibody‐mediated NK‐cell frequencies equal to or above the second quartile (≥ Q2); Specifically, the figures for IFN‐γ‐producing NK‐cell frequencies were 4.73 log_10_ (*p* < 0.005) and 4.54 log_10_ BAU/mL (*p* < 0.005) for Wuhan‐Hu‐1 and Omicron XBB.1.5 (sub)variants, respectively (Figure 5A,C); For LAMP1‐producing NK cell, the frequencies were 4.94 (*p* < 0.005) and 4.75 log_10_ BAU/mL (*p* < 0.005) for Wuhan‐Hu‐1 and Omicron XBB.1.5 (sub)variants, respectively (Figure 5B,D).

## 4 Discussion

Antibodies displaying functional activities against SARS‐CoV‐2, including virus neutralization and mediation of ADCC, that mainly recognize epitopes within the S protein, are thought to critically contribute to virus clearance [4, 5, 6]. Unfortunately, due to their complexity, neither NtAb nor ADCC immunological assays are accessible to most clinical microbiology laboratories. Instead, several quantitative chemiluminescent immunoassays measuring antibody levels (IgGs or total) against the ancestral variant S protein and normalized to the first WHO international standard have been commercialized and are widely used worldwide. It is uncertain whether antibody concentrations measured by these assays may allow estimation of serum/plasma levels of S‐directed antibodies displaying neutralizing or FC‐mediated NK‐cell activities against emerging heterologous SARS‐CoV‐2 Omicron subvariants.

In this proof‐of‐concept study, we used different approaches to determine whether total anti‐RBD antibodies measured by the Roche Elecsys® Anti‐SARS‐CoV‐2 S assay may allow inference of NtAb and Fc‐mediated NK‐cell responses against Omicron XBB.1.5. Even though this subvariant is no longer circulating, its S protein sequence resembles that of currently predominant worldwide circulating JN.1‐derived Omicron (sub)variants, which do not appear to have substantially increased their ability to escape from antibodies elicited by the ancestral strain compared to XBB.1.5 [21], at least when using live virus‐based NtAb assays. We selected elderly nursing home residents for our investigation, as inferring the magnitude of functional antibody responses from data provided by widely available commercial assays could be especially relevant for this population. Such an approach may help assess their risk of severe SARS‐CoV‐2 infection and potentially guide individualized booster vaccination schedules. To our knowledge, no previous studies have addressed this issue. Several main observations were made. First, anti‐RBD total antibody concentrations measured by the Roche assay strongly correlated with NtAb levels against both Wuhan‐Hu‐1 and Omicron XBB.1.5. However, the correlation between anti‐RBD total antibody levels and LAMP1‐ or IFN‐γ‐producing NK‐cell frequencies was moderate for both Wuhan‐Hu‐1 and Omicron XBB.1.5. Taken collectively, the data supported the well‐established assumption that while NtAb antibodies mainly target the RBD sequence, those mediating NK‐cell activity may recognize epitopes mapping outside RBD [4, 5, 6]. In line with our findings, Takei et al. [22] reported a strong linear correlation between antibody levels measured by the Roche assay and NtAb titers against the WK‐521 SARS‐CoV‐2 ancestral strain (Rho = 0.72). Similar findings were reported by Jochum et al. [23] and Taffertshofer et al. [24] using a variety of Wuhan‐Hu‐1‐based virus neutralization assays, including the GenScript cPass™ SARS‐CoV‐2 neutralization antibody detection kit, which is a blocking enzyme‐linked immunosorbent assay intended for the qualitative and semi‐quantitative direct detection of immunoglobulins that neutralize the interaction between RBD and hACE2. Second, Random Forest or random decision machine learning methods have been used to predict negative or positive antibody responses in solid organ transplant recipients following SARS‐CoV‐2 vaccination [25, 26]. Here, we used a comparable approach to assess the ability of the Roche assay to predict NtAb and Fc‐mediated NK‐cell responses against the ancestral variant and Omicron XBB.1.5. Prediction of Wuhan‐Hu‐1 specific NtAb titers from total anti‐RBD levels resulted in mean NRMSEs and NMAEs of 0.24 and 0.18, respectively. Additionally, the model significantly explained data variability (*R* ^2^ = 0.72, 72%). Nevertheless, the lower *R* ^2^ (0.32) observed for the XBB.1.5 variant highlights the challenges in the predictive modeling of functional immune responses against emerging Omicron subvariants. The low‐level circulation of the Omicron XBB.1.5 variant during the study period resulted in a limited dataset, which included many negative samples, compromising the robustness of the Random Forest models for accurate prediction. However, except for NtAb against Omicron XBB.1.5, the remaining predictions showed moderate‐to‐good performance, with average NRMSE and NMAE values below 0.30, and *R* ^2^ values ranging from 0.41 to 0.72. Given that only one predictor variable was used in the modeling, these results suggest that with a more comprehensive dataset reflecting stable variant circulation, such as the Wuhan‐Hu‐1 data, the anti‐RBD antibody levels measured by the Roche assay may have significant predictive potential for both NtAb and Fc‐mediated NK‐cell responses. Identifying easily accessible data with predictive potential for antibody‐mediated immune responses is of great interest. Nevertheless, further research is needed to develop models with greater statistical complexity and accuracy, essential for refining personalized vaccination strategies in at‐risk populations. Finding and integrating other accessible variables with predictive potential—such as demographic or clinical data—could significantly enhance the accuracy of these models. Third, logistic regression modeling allowed us to define anti‐RBD total antibody thresholds predicting detectable and high (≥ Q3) NtAb levels at high probability (> 75%). The anti‐RBD total antibody level threshold predicting detectable NtAb responses against Omicron XBB.1.5 was roughly 3 log_10_ higher than that for Wuhan‐Hu‐1. In contrast, the threshold value differed minimally (around 0.5 log_10_: 5.02 vs. 4.73 BAU/mL) for predicting high‐level NtAb responses, perhaps due to the Omicron XBB.1.5 NtAb data distribution. Notwithstanding, the data further highlighted the potential of Omicron XBB.1.5 to evade NtAb responses elicited by vaccination with Wuhan‐Hu‐1 platforms and natural infection with preceding (sub)variants [7, 8, 9]. Regarding antibody‐mediated NK‐cell frequencies, no anti‐RBD total antibody threshold level predicting high‐level Fc‐mediated NK‐cell responses could be derived from the data. Nevertheless, anti‐RBD total antibody thresholds could be determined to predict antibody‐mediated NK‐cell frequencies equal to or above the second quartile (≥ Q2). Importantly, the figures for both IFN‐γ‐producing and LAMP1‐producing NK‐cell frequencies were comparable for both SARS‐CoV‐2 variants (within the range of 4–5 log_10_ BAU/mL), further suggesting that antibodies mediating NK‐cell functional activities may target relatively conserved epitopes across SARS‐CoV‐2 variants [10, 11, 12, 13, 14]. The main limitation of this study was its small sample size, which precluded subanalyses assessing whether the estimation of functional antibody levels based upon anti‐RBD total antibody concentrations could vary across SARS‐CoV‐2‐naïve and experienced individuals, and among the latter depending on the infecting SARS‐CoV‐2 (sub)variant or vaccination schedules. In summary, antibody concentrations measured by the Roche anti‐RBD total antibody assay may allow inference of NtAb levels, and to a lesser extent Fc‐mediated functional NK‐cell frequencies, directed against Omicron XBB.1.5 in vaccinated elderly nursing home residents.

All in all, this study provides a novel perspective by evaluating the feasibility of inferring functional antibody levels using widely available commercial assays, particularly the Roche Elecsys® Anti‐SARS‐CoV‐2 S assay. This approach addresses critical limitations in the accessibility of neutralizing antibody and Fc‐mediated NK‐cell activity assays, which remain complex and are largely confined to specialized laboratories. While previous studies have reported correlations between commercial immunoassay results and NtAb levels, their applicability has often been constrained by variability across assay types and a predominant focus on the ancestral SARS‐CoV‐2 strain [17, 18, 19, 25, 26]. In contrast, our methodology leverages Random Forest modeling to predict NtAb and NK‐cell responses against both Wuhan‐Hu‐1 and the more immune‐evasive Omicron XBB.1.5 variant, extending the relevance of commercial assay data to emerging subvariants. This framework demonstrates robust predictive power for Wuhan‐Hu‐1 NtAb levels and highlights challenges when extrapolating to new variants, underscoring the need for adaptive modeling strategies and broader datasets for refined prediction accuracy. Thus, our approach offers a pragmatic and scalable solution, potentially informing individualized booster vaccination strategies for at‐risk populations. Thus, further studies are needed to validate our findings and determine whether our observations could be extended to other population groups, particularly those at high risk of developing severe COVID‐19.

## Author Contributions

Design of the study: David Navarro, Ángela Sánchez‐Simarro, and Enric Cuevas‐Ferrando. Acquisition and analysis of data: Ángela Sánchez‐Simarro, Enric Cuevas‐Ferrando, Daniel Fernández‑Soto, Eliseo Albert, Estela Giménez, Ana Isabel Avilés‑Alía, Luciana Rusu, Ron Geller, and Hugh T. Reyburn. Manuscript writing: Ángela Sánchez‐Simarro, Enric Cuevas‐Ferrando, and David Navarro. All authors have read and approved the final manuscript.

## Conflicts of Interest

The authors declare no conflicts of interest.

## Supporting information

Supporting information.

## Data Availability

The data that support the findings of this study are available from the corresponding author upon reasonable request.
