# Supplementary material for: Estimating SARS‐CoV‐2 Omicron XBB.1.5 Spike‐Directed Functional Antibody Levels From an Anti‐Receptor Binding Domain Wuhan‐Hu‐1‐Based Commercial Immunoassay Results
Source: J Med Virol. 2025 Jan 15;97(1):e70130. doi: 10.1002/jmv.70130 (PMC11734093; doi:10.1002/jmv.70130)
Supplement: Supplementary file 1 — Supporting information. [file JMV-97-e70130-s001.docx]

**Estimating SARS-CoV-2 Omicron XBB.1.5 spike-directed functional antibody serum levels from anti-receptor binding domain antibody concentrations measured by a Wuhan-Hu-1-based commercial chemiluminescent immunoassay**

Ángela Sánchez^1^ Enric Cuevas-Ferrando^1^, Daniel Fernández‑Soto^2^, Brayan Grau^3^, Eliseo Albert^1^, Estela Giménez^1,4^, Ana Isabel Avilés‑Alía^3^, Luciana Rusu^3^, Ron Geller^3^, Hugh T. Reyburn^2^ and David Navarro^1,4,5*^

*^1^Microbiology Service, Clinic University Hospital, INCLIVA Health Research Institute, Valencia, Spain.*

*^2^Department of Immunology and Oncology, National Centre for Biotechnology, CNB-CSIC, Madrid, Spain*

*^3^Institute for Integrative Systems Biology (I2SysBio), Universitat de Valencia-CSIC, 46980, Valencia, Spain.*

*^4^Department of Microbiology, School of Medicine, University of Valencia, Valencia, Spain.*

*^5^CIBER de Enfermedades Infecciosas, Instituto de Salud Carlos III, Madrid, Spain.*

**Supplementary material**

**
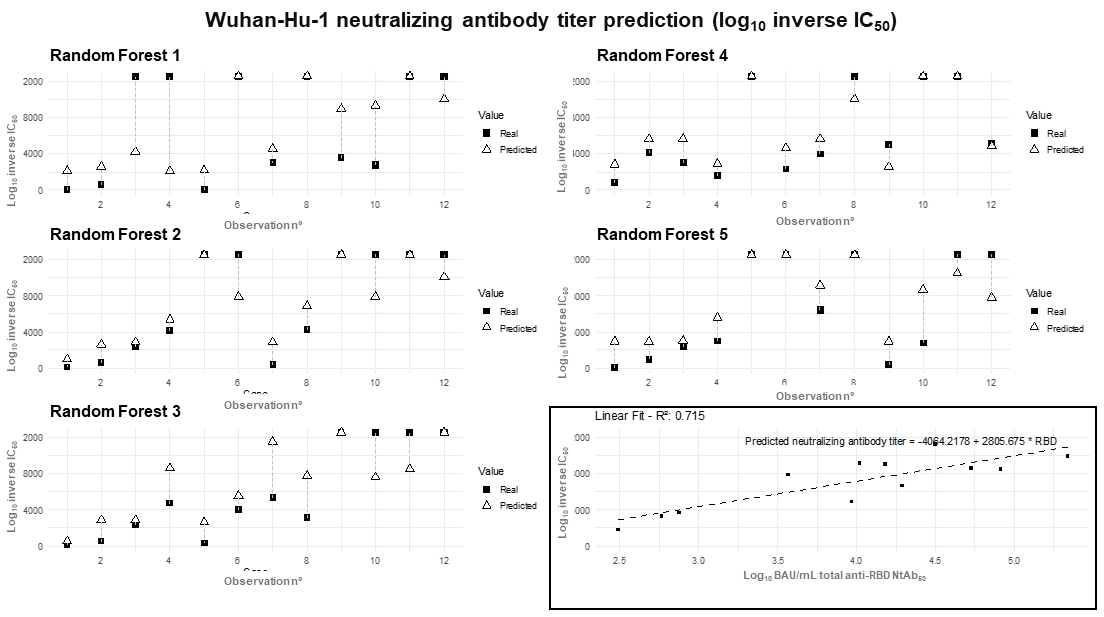
Supplementary Figure 1**


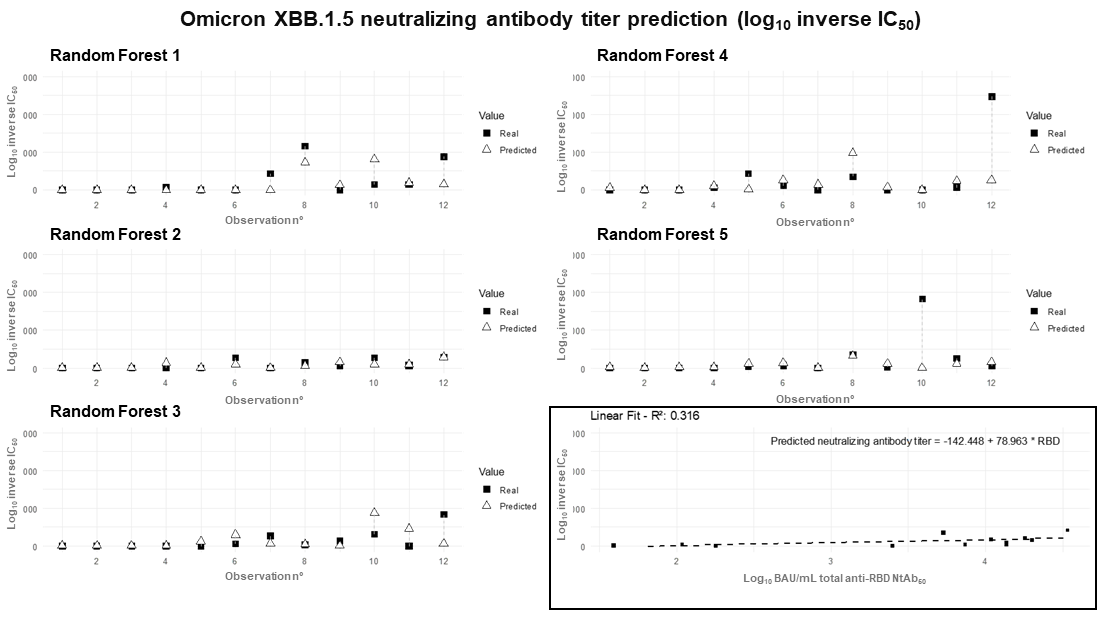
**Supplementary Figure 2**


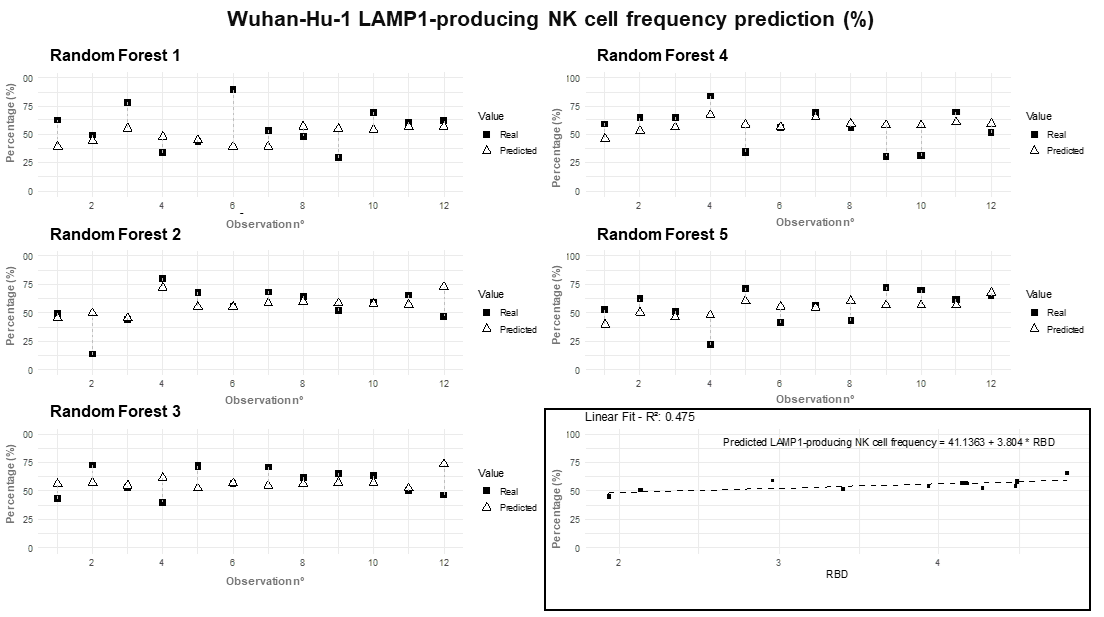
**Supplementary Figure 3**

**Supplementary Figure 4**
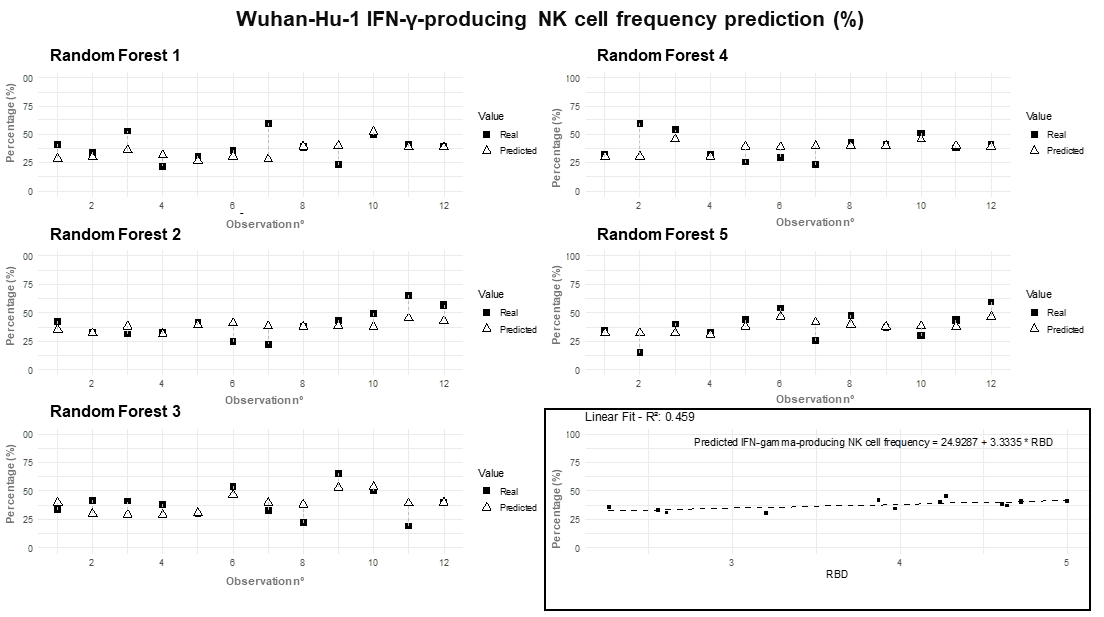


**
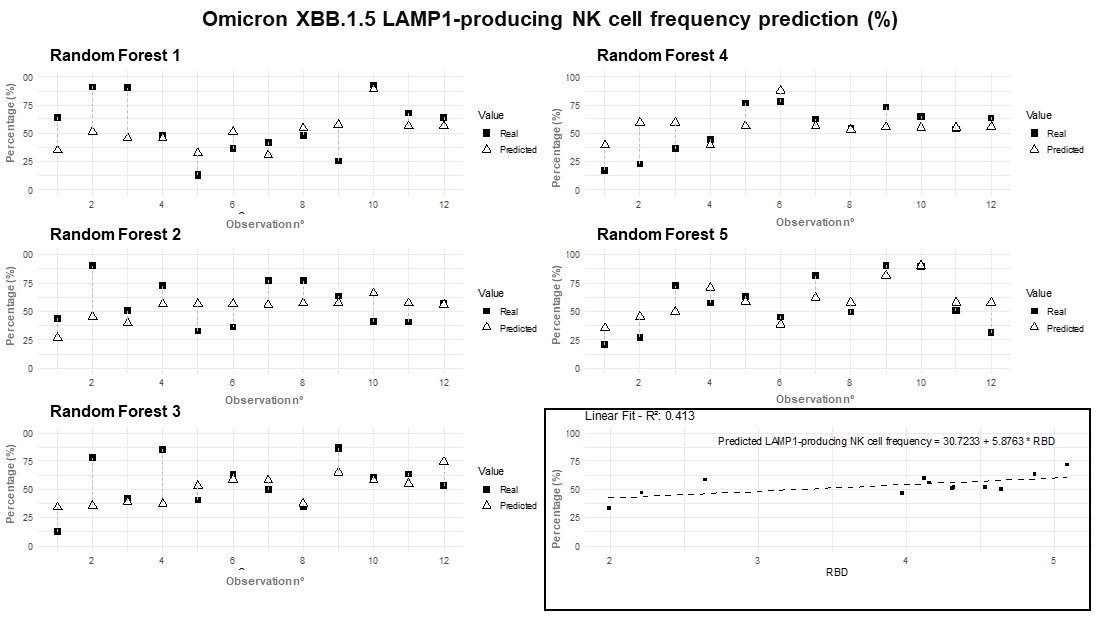
Supplementary Figure 5**

**
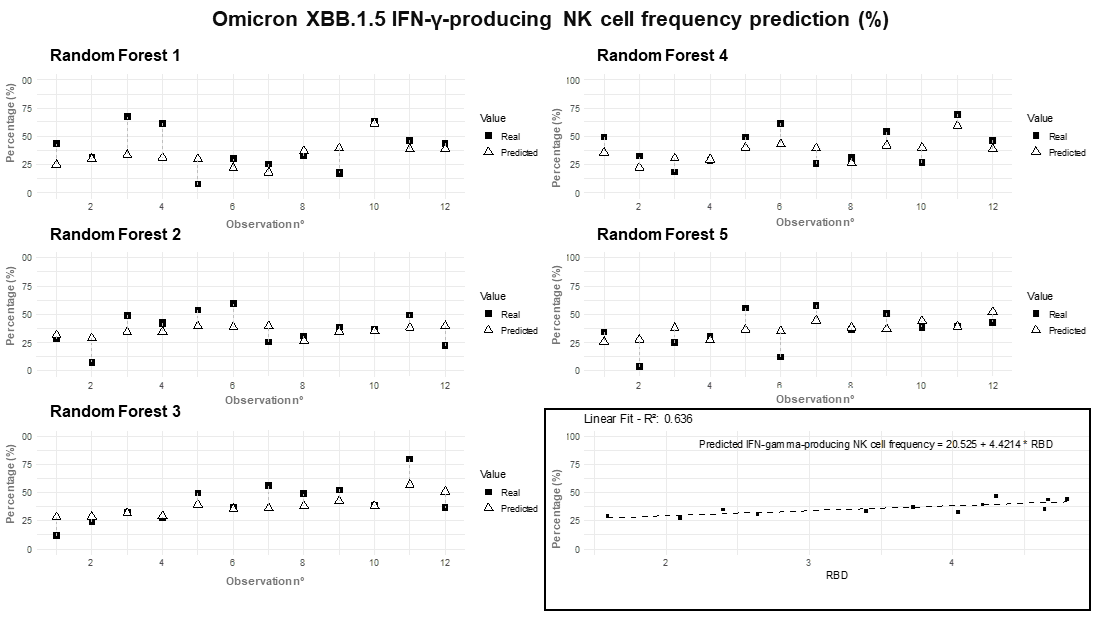
Supplementary Figure 6**

**Figure Legends**

**Supplementary Figure 1.** Random forest models for predicting neutralizing antibody titers (IC_50_) against Wuhan-Hu-1 upon anti-RBD total antibodies measured by the Roche Elecsys® Anti-SARS-CoV-2 S assay and fitted linear regression. Open triangles and black squares indicate actual and predicted values, respectively.

**Supplementary Figure 2.** Random forest models for predicting neutralizing antibody titers (IC_50_) against Omicron XBB.1.5 upon anti-RBD total antibodies measured by the Roche Elecsys® Anti-SARS-CoV-2 S assay and fitted linear regression. Open triangles and black squares indicate actual and predicted values, respectively.

**Supplementary Figure 3.** Random forest models for predicting LAMP1-producing NK cell frequency (%) against Wuhan-Hu-1 upon anti-RBD total antibodies measured by the Roche Elecsys® Anti-SARS-CoV-2 S assay and fitted linear regression. Open triangles and black squares indicate actual and predicted values, respectively.

**Supplementary Figure 4.** Random forest models for predicting IFN-γ-producing NK cell frequency against Wuhan-Hu-1 (%) upon anti-RBD total antibodies measured by the Roche Elecsys® Anti-SARS-CoV-2 S assay and fitted linear regression. Open triangles and black squares indicate actual and predicted values, respectively.

**Supplementary Figure 5.** Random forest models for predicting LAMP1-producing NK cell frequency (%) against Omicron XBB.1.5 upon anti-RBD total antibodies measured by the Roche Elecsys® Anti-SARS-CoV-2 S assay and fitted linear regression. Open triangles and black squares indicate actual and predicted values, respectively.

**Supplementary Figure 6.** Random forest models for predicting IFN-γ-producing NK cell frequency (%) against Omicron XBB.1.5 upon anti-RBD total antibodies measured by the Roche Elecsys® Anti-SARS-CoV-2 S assay and fitted linear regression. Open triangles and black squares indicate actual and predicted values, respectively.
